# Chlorophyll Catabolites in Senescent Leaves of the Plum Tree (*Prunus domestica*)

**DOI:** 10.1002/cbdv.201600181

**Published:** 2016-11-07

**Authors:** Theresia Erhart, Cecilia Mittelberger, Clemens Vergeiner, Gerhard Scherzer, Barbara Holzner, Peter Robatscher, Michael Oberhuber, Bernhard Kräutler

**Affiliations:** ^1^ Institute of Organic Chemistry and Center of Molecular BiosciencesUniversity of InnsbruckInnrain 80/82AT‐6020Innsbruck; ^2^ Laimburg Research Centre for Agriculture and ForestryLaimburg 6 – Pfatten (Vadena)IT‐39040Auer (Ora)BZ

**Keywords:** Chlorophyll, Fruit, Phyllobilins, Porphyrins, Plant senescence

## Abstract

In cold extracts of senescent leaves of the plum tree (*Prunus domestica* ssp. *domestica*), six colorless non‐fluorescent chlorophyll catabolites (NCCs) were characterized, named *Pd*‐NCCs. In addition, several minor NCC fractions were tentatively classified. The structure of the most polar one of the NCCs, named *Pd*‐NCC‐32, featured an unprecedented twofold glycosidation pattern. Three of the NCCs are also functionalized at their 3^2^‐position by a glucopyranosyl group. In addition, two of these glycosidated NCCs carry a dihydroxyethyl group at their 18‐position. In the polar *Pd*‐NCC‐32, the latter group is further glycosidated at the terminal 18^2^‐position. Four other major *Pd*‐NCCs and one minor *Pd*‐NCC were identified with five NCCs from higher plants known to belong to the ‘*epi*’‐series. In addition, tentative structures were derived for two minor fractions, classified as yellow chlorophyll catabolites, which represented (formal) oxidation products of two of the observed *Pd*‐NCCs. The chlorophyll catabolites in leaves of plum feature the same basic structural pattern as those found in leaves of apple and pear trees.

## Introduction

**Figure 1:**
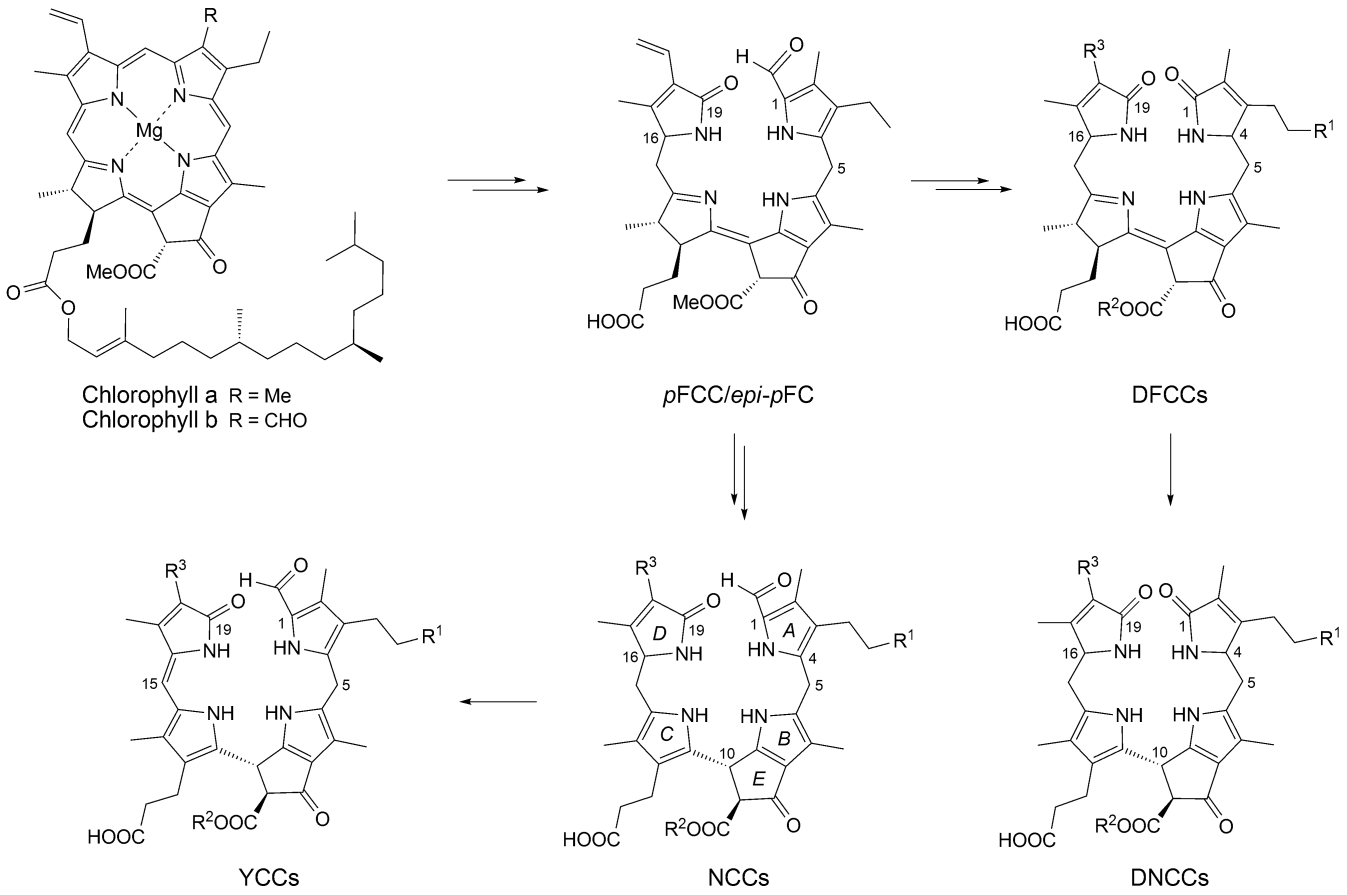
Short outline of the main path of chlorophyll breakdown in higher plants, displaying structural formulas of chlorophylls *a* and *b*, of the primary fluorescent chlorophyll catabolites (*p*FCC/*epi*‐*p*FCC), of non‐fluorescent chlorophyll catabolites (NCCs), of yellow chlorophyll catabolites (YCCs), of dioxobilin‐type FCCs (DFCCs) and of dioxobilin‐type NCCs (DNCCs) (generalized formulas, see *Table* 1 for individual NCCs^8^, ^9^).

About 25 years ago, chlorophyll (Chl) breakdown and the appearance of the fall colors were still a stunning mystery.^1^, ^2^ In 1991, a first colorless Chl degradation product from a higher plant was described, the ‘non‐fluorescent’ Chl‐catabolite (NCC) *Hv*‐NCC‐1 from senescent leaves of barley (*Hordeum vulgare*).^3^, ^4^ Structural identification of *Hv*‐NCC‐1 as a 1‐formyl‐19‐oxobilin‐type linear tetrapyrrole^3^ opened the door to the structure‐guided discovery of the ‘PaO/phyllobilin’ pathway of Chl‐breakdown.^5^, ^6^, ^7^, ^8^, ^9^ As we know now, oxidative cleavage of the Chl macroring generates 1‐formyl‐19‐oxobilins and sets the stage for the formation of various bilin‐type catabolites of Chl,^10^, ^11^ or ‘phyllobilins’.^7^, ^8^, ^9^ The ‘early’ stages of Chl‐breakdown, which take place in the chloroplasts, furnish one of two epimeric primary ‘fluorescent’ Chl‐catabolites (*p*FCCs), with species‐dependent configuration of their formation.^11^, ^12^ *p*FCCs are rapidly hydroxylated to 3^2^‐OH‐*p*FCC (probably still in the chloroplast).^7^ Once exported into the cytosol, FCCs are mostly modified further and imported into the acidic vacuoles, where they are thought to isomerize spontaneously to corresponding NCCs (see *Fig.* 1).^13^

In the meantime, NCCs have been found in extracts of senescent leaves of a range of plants,^5^, ^6^, ^7^, ^8^, ^9^, ^14^, ^15^ where they accumulate and were suggested earlier to represent ‘final stages’ of Chl‐catabolism.^5^, ^6^, ^16^ NCCs were also identified as products of Chl breakdown in ripening fruit^17^, ^18^, ^19^, ^20^ and in de‐greening vegetables.^21^, ^22^, ^23^ In the last 25 years, more than 20 structurally different NCCs from higher plants were, thus, detected, and their structures were characterized (see *Table* 1).^8^, ^9^, ^14^, ^24^ Evidence for further oxidative transformation of NCCs in leaves was also provided more recently by the observation of yellow Chl‐catabolites (YCCs)^25^, ^26^, ^27^, ^28^ and pink Chl‐catabolites (PiCCs)^29^ in senescent leaves of a variety of higher plants.^9^ These colored phyllobilins were identified as formal dehydrogenation products of corresponding tetrapyrrolic NCCs. All of these observations were consistent with an essentially ‘linear’ path of Chl‐breakdown in higher plants.^5^, ^6^, ^7^

**Table 1:** Structures of known natural nonfluorescent Chl‐catabolites (NCCs)

R^1^	R^2^	R^3^	C(16)^a^	Provisional names^b^	Ref.
H	H	CH=CH_2_	n	*Bo*‐NCC‐2 (*At*‐NCC‐3^c^)	23 30
H	Me	CH=CH_2_	*epi*	*Cj*‐NCC‐2 (*So*‐NCC‐5/***Pd*** **‐NCC‐71** d)	13 22
OH	H	CH=CH_2_	n	*Bn*‐NCC‐3	31
OH	H	CH=CH_2_	*epi*	*So*‐NCC‐3 (*Mc*‐NCC‐49)	18 22
OH	H	CH(OH)–CH_2_OH	*epi*	*So*‐NCC‐1 (*Mc*‐NCC‐26)	18 22
OH	Me	CH=CH_2_	n	*Sw*‐NCC‐58	32
OH	Me	CH=CH_2_	*epi*	*Cj*‐NCC‐1 (*So*‐NCC‐4/*Md*‐NCC‐2/***Pd*** **‐NCC‐60** d)	13 17 22 33
OH	Me	CH(OH)‐CH_2_OH	n	*Hv*‐NCC‐1	3 4
OH	Me	CH(OH)–CH_2_OH	*epi*	*So*‐NCC‐2 (*Mc*‐NCC‐42/***Pd*** **‐NCC‐40** d ^,^ e)	18 21 22
O‐Glc	H	CH=CH_2_	n	*Bn*‐NCC‐2 (*At*‐NCC‐1^e^/*Bo*‐NCC‐1)	15 23 31
O‐Glc	H	CH=CH_2_	*epi*	*Co*‐NCC‐2^e^	20
O‐Glc	Me	CH=CH_2_	n	*At‐*NCC‐4^e^	15
O‐Glc	Me	CH=CH_2_	*epi*	*Nr*‐NCC‐2 (*Md*‐NCC‐1/***Pd*** **‐NCC‐56** d ^,^ e)	17 34 35
O‐Glc	Me	CH(OH)–CH_2_OH	*epi*	*Zm*‐NCC‐1 (*Tc*‐NCC‐1/***Pd*** **‐NCC‐35** d ^,^ e)	26 35
O‐Glc	Me	CH(OH)–CH_2_O‐Glc	*epi*	***Pd‐*** **NCC‐32** d	
O‐(6′‐O‐Mal)Glc	Me	CH=CH_2_	*epi*	*Nr*‐NCC‐1	34
O‐Mal	H	CH=CH_2_	n	*Bn*‐NCC‐1	31 36
O‐Mal	Me	CH=CH_2_	*epi*	*Ej*‐NCC‐2^e^	19
O‐Glc^f^	Me	CH=CH_2_	*epi*	*Ug*‐NCC‐53	37

Mal, malonyl; Glc, *β*‐glucopyranosyl. ^a^ Configuration at C(16); NCCs derived from *p*FCC (n, ‘normal’) or from *epi‐p*FCC (*epi*, ‘epimeric’), the absolute configuration at C(16) is not determined. ^b^ *Bo*‐NCCs (from broccoli, *Brassica oleracea* var. *italica*),^23^ *At*‐NCCs (from *Arabidopsis thaliana*),^30^, ^15^ *Cj*‐NCCs (from Katsura tree, *Cercidiphyllum japonicum*),^13^, ^33^ *So*‐NCCs (from spinach, *Spinacia oleracea*),^21^, ^22^ *Bn*‐NCCs (from oilseed rape, *Brassica napus*),^31^, ^36^ *Mc*‐NCCs (from banana peels, *Musa acuminate*, Cavendish cultivar),^18^ *Sw*‐NCC‐58 (from Peace Lily, *Spathiphyllum wallisii*),^32^ *Md*‐NCCs (from *Malus domestica*),^17^ *Hv*‐NCC‐1 (from barley, *Hordeum vulgare*),^3^, ^4^ *Co*‐NCC‐2 (from quince fruits, *Cydonia oblonga*),^20^ *Nr*‐NCCs (from tobacco, *Nicotiana rustica*),^34^ *Zm*‐NCC‐1 (from maize, *Zea mays*),^35^ *Tc*‐NCC‐1 (from Lime tree, *Tilia cordata*),^26^ *Pd*‐NCCs are from this work and are shown in bold (from Plum tree, *Prunus domestica*), *Ej*‐NCC‐2 (from loquat fruits, *Eriobotrya japonica*)^19^ and *Ug*‐NCC‐53 (from Wych Elm tree, *Ulmus glabra*).^37^ ^c^ *At*‐NCC‐3 carries a HOCH_2_ group at C(2).^30^ ^d^ This work. ^e^ Structure assigned tentatively based on UV/VIS and mass spectra. ^f^ The *β*‐glucopyranosyl group attached at C(3^2^) is also esterified with its primary OH group at the propionate function, giving a bicyclo[17.3.1] motif.^37^

However, as was recognized recently, Chl‐breakdown ‘branches out’, and furnishes ‘1,19‐dioxobilin‐type’ Chl‐catabolites (DCCs)^38^ as second major family of phyllobilins.^7^, ^8^, ^9^ The latter (‘type‐II’) phyllobilins are mostly colorless, such as the 1,19‐dioxobilin‐type NCCs (DNCCs).^9^, ^39^, ^40^ Originally, DNCCs were suggested to be oxidative deformylation products of NCCs.^38^ In view of a surprising stereochemical diversity observed in natural DNCCs, we suggested an earlier branching‐point in the ‘PaO/phyllobilin’ pathway of Chl‐breakdown.^9^, ^39^ Indeed, a P450‐enzyme catalyzing deformylation of FCCs was identified,^40^ which converts FCCs (1‐formyl‐19‐oxobilin‐type or ‘type‐I’ phyllobilins) to corresponding 1,19‐dioxobilin‐type FCCs (DFCCs), hence, opening the pathway to ‘type‐II’ phyllobilins.^9^, ^40^, ^41^ Under weakly acidic conditions, the latter are indicated to isomerize stereoselectively to corresponding DNCCs (see *Fig.* 1).^41^

In the context of investigations of Chl‐catabolites in domestic agricultural plants, we have studied the nature of such phyllobilins in stone fruit and report here our work on the Chl‐catabolites in leaves of the plum tree (*Prunus domestica* ssp. *domestica*). As shown below, Chl‐breakdown in senescent leaves of this fruit tree follows the ‘PaO/phyllobilin’ pathway of Chl‐breakdown.^7^, ^8^, ^9^ It produces 1‐formyl‐19‐oxobilin‐type catabolites, or ‘type‐I’ phyllobilins, identified as NCCs of the ‘*epi*‐type’. In addition, in the extracts several YCCs were also found.

## Results and Discussion

**Figure 2:**
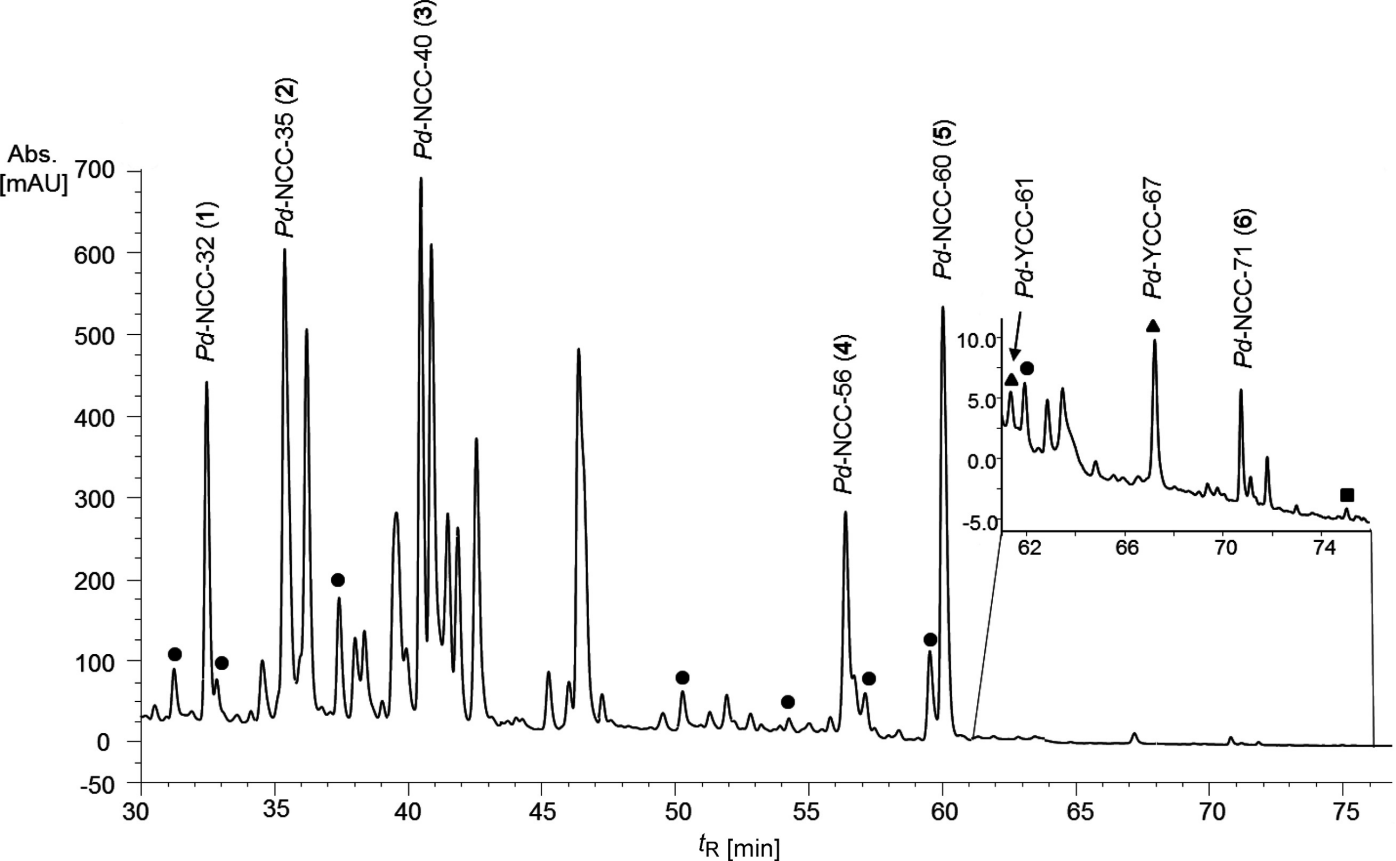
HPLC Analysis of an extract of senescent plum tree (*Prunus domestica*) leaves (online detection at 320 nm). Main catabolites are highlighted by standard names of catabolites; minor fractions classified as ● non‐fluorescent chlorophyll catabolites (NCC), ▲ yellow chlorophyll catabolites (YCC) and ■ pink chlorophyll catabolites (PiCC), based on their UV/VIS spectra (see text for details).

Yellow senescent and green leaves were collected from plum trees (*Prunus domestica*) and frozen for storage. Five major and nine minor colorless NCCs were provisionally identified in extracts of senescent leaves of plum trees on the basis of their characteristic UV‐absorbance properties, using analytical HPLC (*Fig.* 2). All NCC fractions showed UV/VIS spectra featuring a longest wavelength maximum near 314 nm, characteristic of an *α*‐formyl‐pyrrole moiety (ring *A*), as first reported in the spectrum of *Hv*‐NCC‐1.^3^ Likewise, minor fractions of two YCCs and a trace of a pink Chl‐catabolite (PiCC) were also tentatively identified.

**Figure 3:**
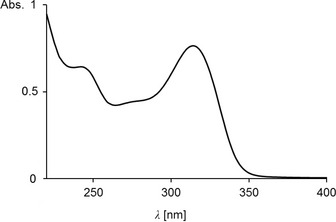
UV/VIS Spectrum of *Pd*‐NCC‐32 (**1**) in MeOH (*c* = 4.3 × 10^−5^ m).

For spectroscopic analysis of the most abundant NCCs in the leaves of *P*. *domestica*, 18.7 g of senescent plum tree leaves were extracted with cold MeOH (to avoid significant NCC oxidation, see 28), and the extract was separated by semi‐preparative HPLC. A two‐stage purification procedure gave a uniform sample of 0.29 mg of *Pd*‐NCC‐32 (**1**), analyzed by UV/VIS‐spectroscopy first (see *Fig.* 3). CD Spectra of *Pd*‐NCC‐32 (**1**) and of *Hv*‐NCC‐1^4^ showed the same basic features, suggesting a common (*R*)‐configuration at the stereogenic C(10).^9^

**Figure 4:**
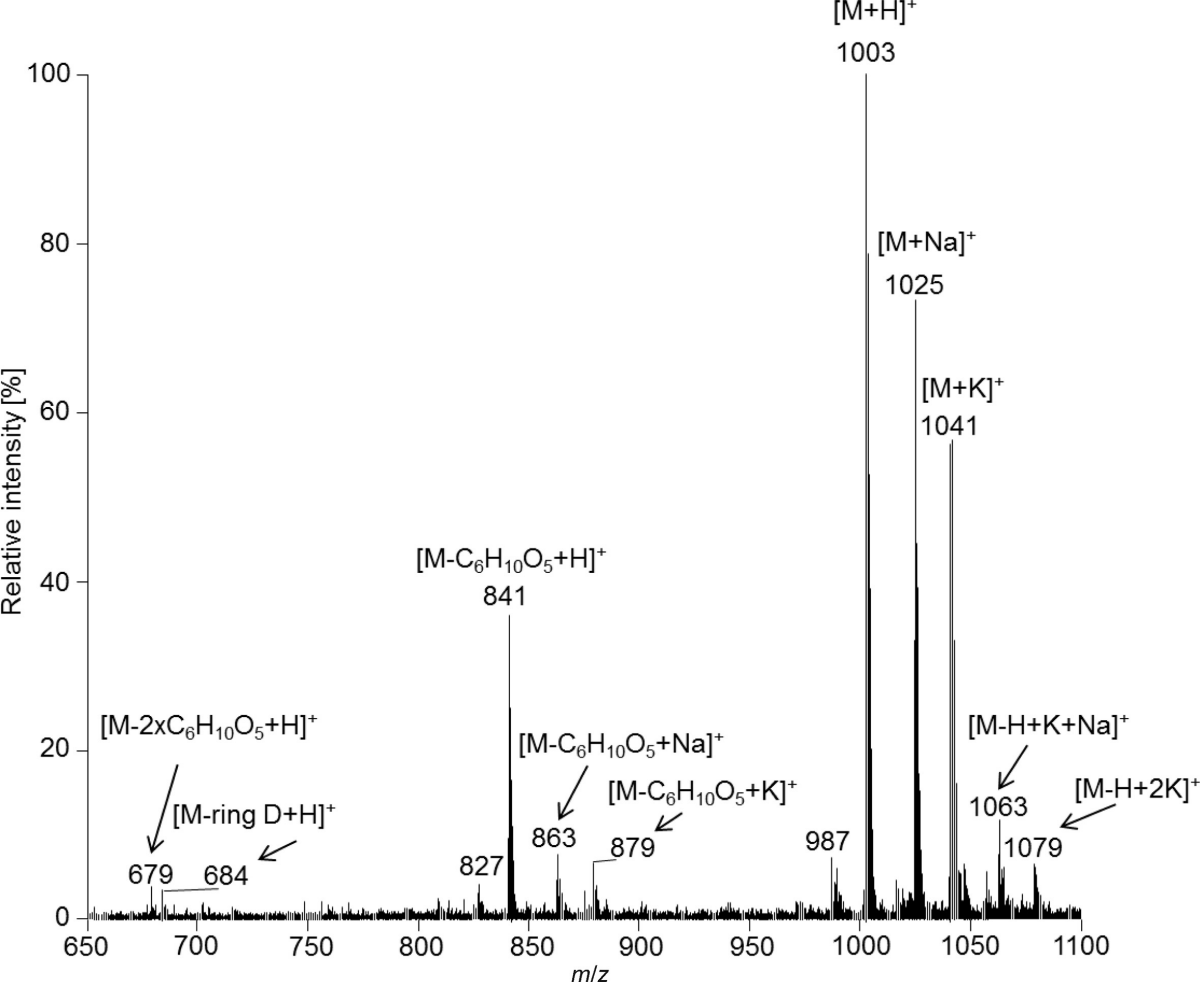
Electrospray ionization mass spectrum of *Pd*‐NCC‐32 (**1**) in the positive‐ion mode.

A high resolution MALDI‐MS spectrum of *Pd*‐NCC‐32 (**1**) displayed a strong signal at *m*/*z* 1025.3839, corresponding to $C_{47}H_{62}N_{4}{\text{NaO}}_{20}^{+}$ ([*M* + Na]^+^, calc. 1025.3850) and establishing the molecular formula as C_47_H_62_N_4_O_20_. Likewise, a positive‐ion‐mode ESI‐MS spectrum^42^ (see *Fig.* 4) displayed its *pseudo*‐molecular ion [*M* + H]^+^ at *m*/*z* 1003.1, also consistent with the molecular formula of C_47_H_62_N_4_O_20_. Characteristic fragment ions at *m*/*z* 841.2, 684.1 and 679.2 were also detected, which indicated the loss of a sugar moiety (C_6_H_10_O_5_) from [*M* + H]^+^,^31^ the loss of ring *D* (see 42) or of a second sugar moiety, respectively.

**Figure 5:**
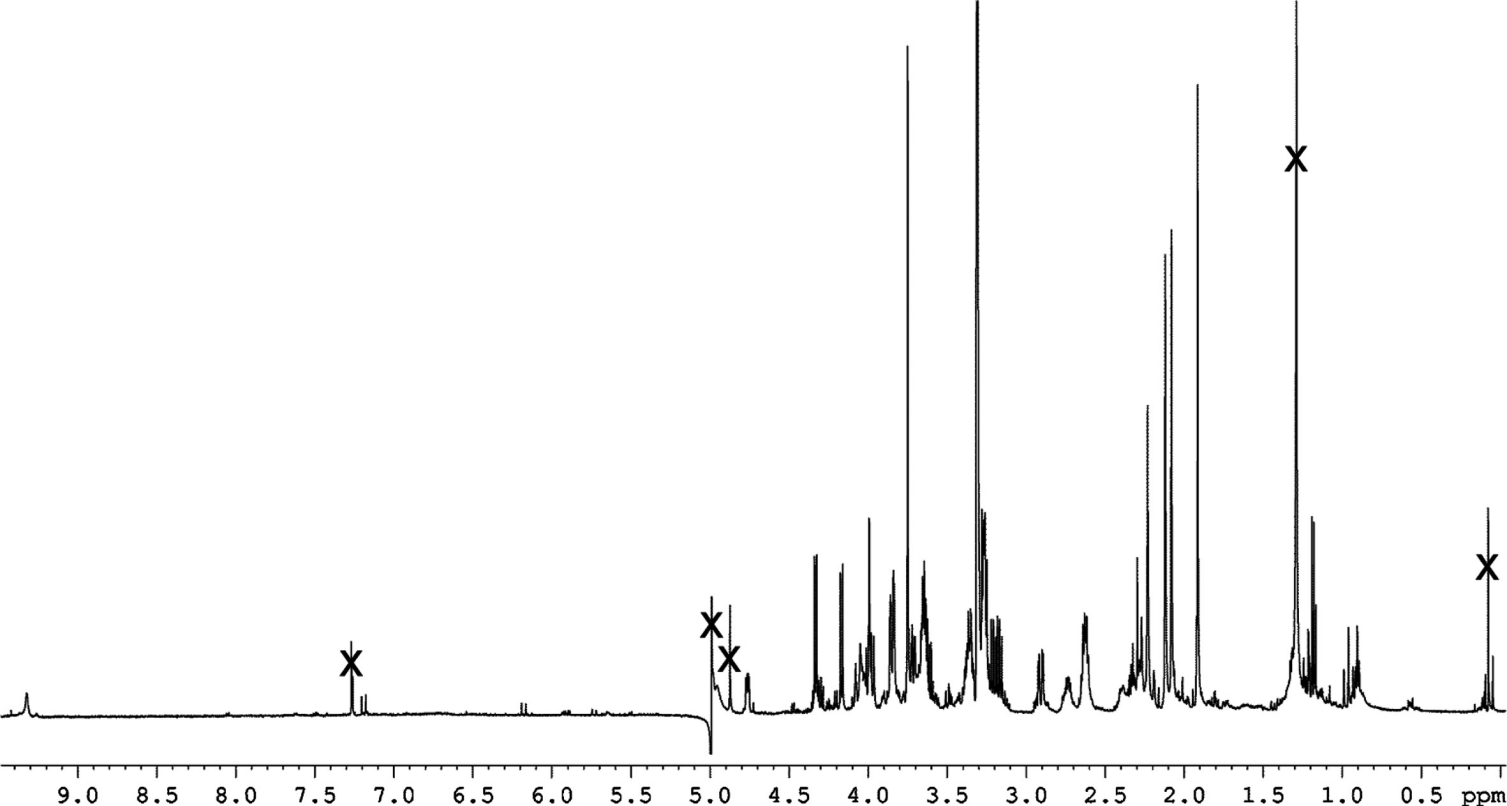
^1^H‐NMR (600 MHz) Spectrum of *Pd*‐NCC‐32 (**1**) in CD_3_ OD (10 °C, ‘×’ marks solvent signals).

**Figure 6:**
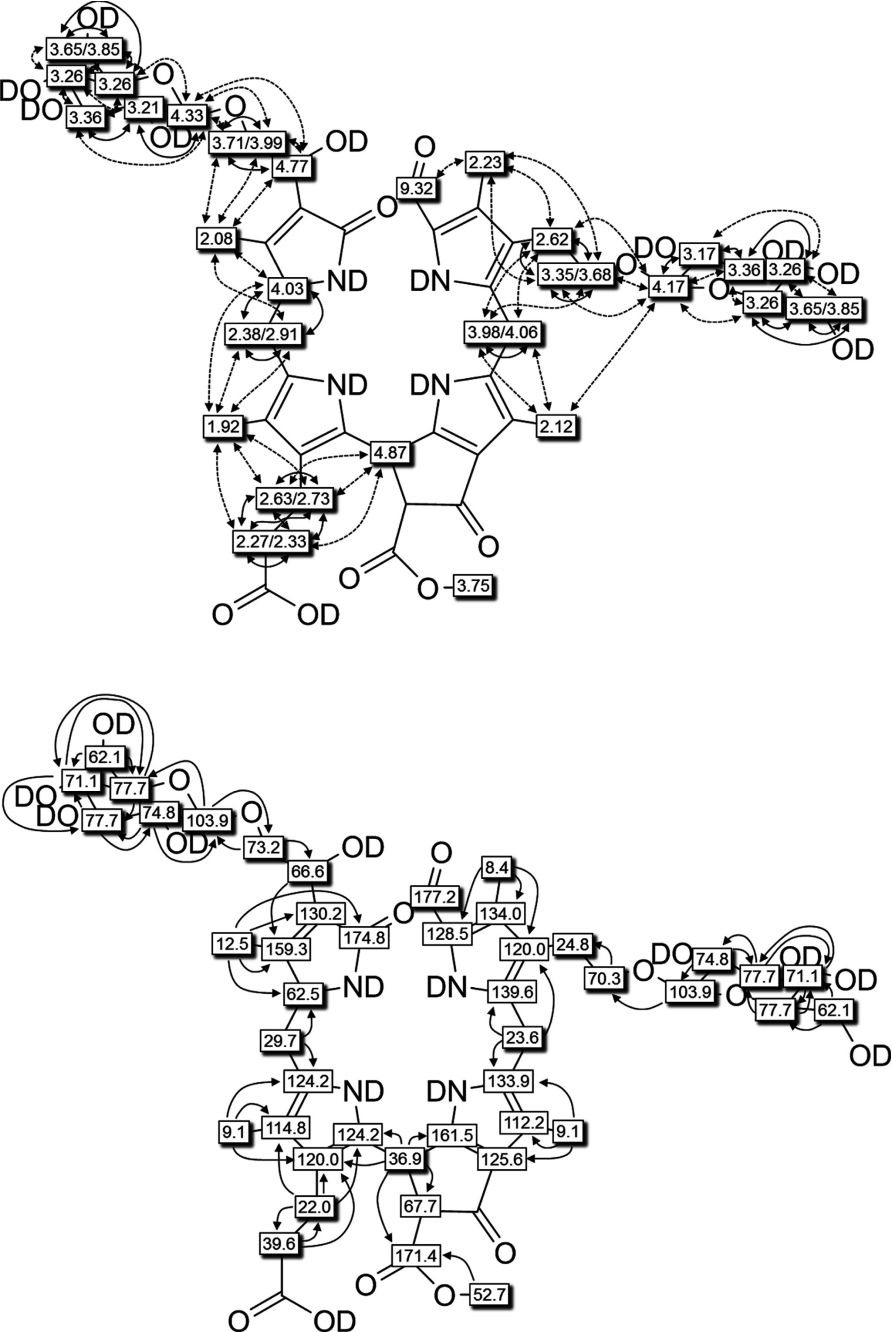
Graphical structural analysis of *Pd‐* NCC‐32 (**1**) based on NMR (600 MHz) spectra (in CD_3_ OD, 283 K). Top: ^1^H‐chemical shift assignments from ^1^H,^1^H‐ROESY and ^1^H,^1^H‐COSY correlations (dashed or solid arrows, respectively). Bottom: ^13^C chemical assignments based on direct ^1^H,^13^C‐HSQCs (shaded boxes) and on ^1^H,^13^C‐HMBCs (symbolized by arrows, open boxes).

In a 600 MHz ^1^H‐NMR spectrum of *Pd*‐NCC‐32 (**1**) in CD_3_OD at 10 °C (see *Fig.* 5) signals of 47 of the 48 C‐bound H‐atoms were observed. Among these signals there were a *singlet* for the formyl H‐atom (H–C(20)) at low field, four Me group *singlet*s at high field and a *singlet* for the methyl ester group at 3.75 ppm. The typical signals for a peripheral vinyl group were not observed. From ^1^H,^13^C‐heteronuclear (HSQC and HMBC) and ^1^H,^1^H‐homonuclear NMR‐correlations (COSY and ROESY) of *Pd*‐NCC‐32 (**1**) in CD_3_OD, assignment of the signals of 47 H‐atoms and 45 ^13^C‐nuclei could be achieved (see *Fig.* 6). In addition to the signals of the NCC‐core, those of 14 H‐atoms were observed in the intermediate field of the ^1^H‐NMR spectrum. ^1^H,^1^H‐COSY and ^1^H,^13^C‐HSQC correlations indicated two hexopyranose units, with closely similar ^1^H‐ and ^13^C‐shifts in both sugar moieties. Only for atoms at or close to the anomeric centre, H–C(1′) (4.17 ppm) and H–C(1″) (4.33 ppm), as well as H–C(2′) (3.17 ppm) and H–C(2″) (3.21 ppm), the chemical shifts of the pairs of signals differed significantly (for atom numbering: see Experimental Section, *Fig*. 9). Chemical shifts and *doublet* nature (*J* = 7.8 Hz) of H–C(1′) and H–C(1″) indicated *β*‐anomeric attachment of both sugar moieties, as observed earlier for the 3^2^‐glucopyranoside moieties of NCCs.^26^, ^31^, ^34^, ^35^ Indeed, both sugar units were identified as glucopyranosides by comparing the ^1^H‐ and ^13^C‐chemical shifts of *Pd*‐NCC‐32 (**1**) with those of the known NCCs with a peripheral glucopyranosyl group at C(3^2^).^26^, ^31^, ^34^, ^35^ ^1^H,^13^C‐HMBCs from H–C(1′) with C(3^2^) and from H–C(1″) with C(18^2^) established the attachment of one sugar moiety at each one of the terminal C‐atoms of the Et side chain at C(3) (ring *A*) and of the 1,2‐dihydroxyethyl group at C(18) (ring *D*). The ^1^H‐ and ^13^C‐chemical shifts at the positions C(18^2^) and C(3^2^) were also consistent with an attached peripheral sugar substituent. However, as with other 1,2‐dihydroxyethyl substituted NCCs,^3^, ^4^, ^7^, ^9^ in **1** the configuration at C(18^2^) remains unknown.

**Figure 7:**
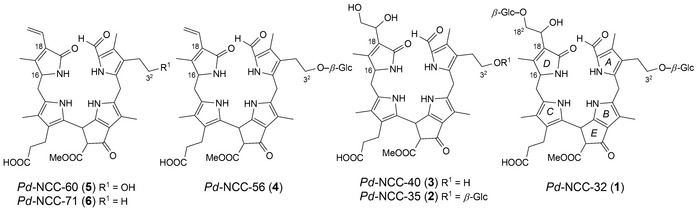
Constitutional formulas of non‐fluorescent chlorophyll catabolites (NCCs) found in senescent leaves of the plum tree (*P*. *domestica* ssp. *domestica*).

Five other *Pd*‐NCC fractions (see *Fig.* 2), *i.e*., *Pd*‐NCC‐35 (**2**), *Pd*‐NCC‐40 (**3**), *Pd*‐NCC‐56 (**4**), *Pd*‐NCC‐60 (**5**), and *Pd*‐NCC‐71 (**6**) were also isolated and purified by HPLC. A positive‐ion‐mode ESI‐MS spectrum of *Pd*‐NCC‐60 (**5**) showed a *pseudo*‐molecular ion [*M* + H]^+^ at *m*/*z* 645.2, consistent with the molecular formula of C_35_H_40_N_4_O_8_. Characteristic fragment ion peaks were visible at *m*/*z* 613.2 and 522.1, corresponding to the loss of MeOH and the loss of ring *D* from [*M* + H]^+^. The same molecular formula and fragmentation is known for the major NCC from *Cercidiphyllum japonicum* (*Cj*‐NCC‐1),^33^ an abundant NCC with ‘*epi*’‐configuration at C(16).^9^, ^21^ To test the probable identity of these two NCCs, their elution properties were compared in HPLC experiments. Thus, solutions of *Pd*‐NCC‐60 (**5**) and *Cj*‐NCC‐1 were separately analyzed by analytical HPLC, as well as a 1:1 mixture of both in a co‐injection (see Experimental Section, *Fig*. 10). Their common elution time and their common UV/VIS‐ and mass spectral data, suggest structural identity of the NCCs *Cj*‐NCC‐1 and *Pd*‐NCC‐60 (**5**) (see *Fig.* 7), implying *‘epi’*‐configuration at C(16) of *Pd*‐NCC‐60 (**5**). Consistent with their origin from a common ‘primary’ FCC,^5^, ^6^, ^7^, ^8^, ^9^ now indicated to be *epi*‐*p*FCC, the other colorless *Pd*‐NCCs were also deduced to belong to the *epi*‐series.

The molecular formula of *Pd*‐NCC‐56 (**4**) was determined as C_41_H_50_N_4_O_13_ by ESI mass spectrometry, which furnished a base peak [*M* + H]^+^ at *m*/*z* 807.2. Fragment‐ions at *m*/*z* 775.3, 684.2 and 645.2 corresponded to the loss, alternatively, of MeOH, of ring *D* and of a hexose moiety from [*M* + H]^+^. These data indicate the presence of one hexopyranose moiety at the HO–C(3^2^) group of ring *A* of *Pd*‐NCC‐56 (**4**) and a vinyl group at C(18) of ring *D*. This indicates a common chemical constitution of **4** and of *Nr*‐NCC‐2^34^ (see *Fig.* 7).

The molecular formula of *Pd*‐NCC‐40 (**3**) could be deduced tentatively as C_35_H_42_N_4_O_10_ by ESI mass spectrometry, which showed the experimental base peak [*M* + H]^+^ at *m*/*z* 679.2. In the mass spectra, characteristic fragment‐ion peaks at *m*/*z* 647.2 and 522.1 were also detected, which corresponded to the loss of MeOH and to the loss of ring *D* (from [*M* + H]^+^). Accordingly, the catabolite *Pd*‐NCC‐40 (**3**) (see *Fig.* 7) was deduced to have the same chemical constitution as *So*‐NCC‐2 from spinach.^21^, ^22^

A positive‐ion‐mode ESI‐MS spectrum of *Pd*‐NCC‐35 (**2**) indicated a *pseudo*‐molecular ion at *m*/*z* 841.2, consistent with the molecular formula of C_41_H_52_N_4_O_15_. The fragments at *m*/*z* 809.3, 684.2, 679.2 and 522.1 indicated the loss of MeOH, the loss of ring *D*, the loss of a sugar moiety and the loss of ring *D* and a sugar moiety. Thus, the catabolite **2** carries a sugar substituent at the C(3) hydroxyethyl side chain (ring *A*) and a 1,2‐dihydroxyethyl group at C(18) (ring *D*). According to their fragmentation pattern,^42^ *Pd*‐NCC‐35 (**2**) (see *Fig.* 7) and *Zm*‐NCC‐1^35^ show the same chemical constitution.

The molecular formula of *Pd*‐NCC‐71 (**6**) was determined as C_35_H_40_N_4_O_7_ with a *pseudo*‐molecular ion at *m*/*z* 629.2. Fragments at *m*/*z* 597.2 and 506 indicate the loss of MeOH and ring *D*. *Pseudo*‐molecular ion and fragment‐ions are consistent with a chemical constitution of **6**, as previously found for *Cj*‐NCC‐2 (*Fig.* 7).^13^ Identity of *Pd*‐NCC‐71 (**6**) and of *Cj*‐NCC‐2 was supported by a common retention time of **6** and *Cj*‐NCC‐2 in a HPLC co‐injection experiment.

Analysis of a minor NCC (tentatively named *Pd*‐NCC‐54) by LC/ESI‐MS revealed a *pseudo*‐molecular ion at *m*/*z* 661.2 ([*M* + H]^+^), consistent with the molecular formula of C_35_H_40_N_4_O_9_. We suspected *Pd*‐NCC‐54 as product of the formal addition of an O‐atom to *Pd*‐NCC‐60 (**5**) from an endogenous oxidation process. Indeed, as shown recently,^28^ NCCs may undergo C(15) hydroxylation by endogenous, as well as by additional efficient adventitious oxidation during preparation of leaf homogenates and their extracts. From NCCs hydroxylated at their C(15) position, H_2_O may eliminate easily, resulting in corresponding YCCs.^28^ Indeed, a YCC was detected in the fresh plum leaf extracts, named *Pd*‐YCC‐67, which showed mass spectral data (*pseudo*‐molecular ion with *m*/*z* 643.2) consistent with its formation as the formal product of an oxidative dehydrogenation of *Pd*‐NCC‐60 (**5**). A further minor fraction, classified as YCC from a prominent absorption maximum near 420 nm, was also subjected further to ESI‐MS analysis. The latter data suggested *Pd*‐YCC‐61 (*m*/*z* 805.1) to represent a YCC derived from oxidation of the glucosylated *Pd*‐NCC‐56 (**4**). When extracts were prepared after storage of senescent leaves of the plum tree at room temperature for 7 min, an increase of the content of both YCCs (*Pd*‐YCC‐61 and *Pd*‐YCC‐67) was observed, as well as the formation of 15‐OH‐*Pd*‐NCC‐60, identified by comparison with its analogue from the established oxidation of *Cj*‐NCC‐1.^28^ However, this hydroxylated NCC differed (in its retention time) from *Pd*‐NCC‐54. Clearly, work‐up and preparation of extracts of cold senescent leaves need to be done swiftly, in order to avoid oxidation artefacts.

## Conclusions

**Figure 8:**
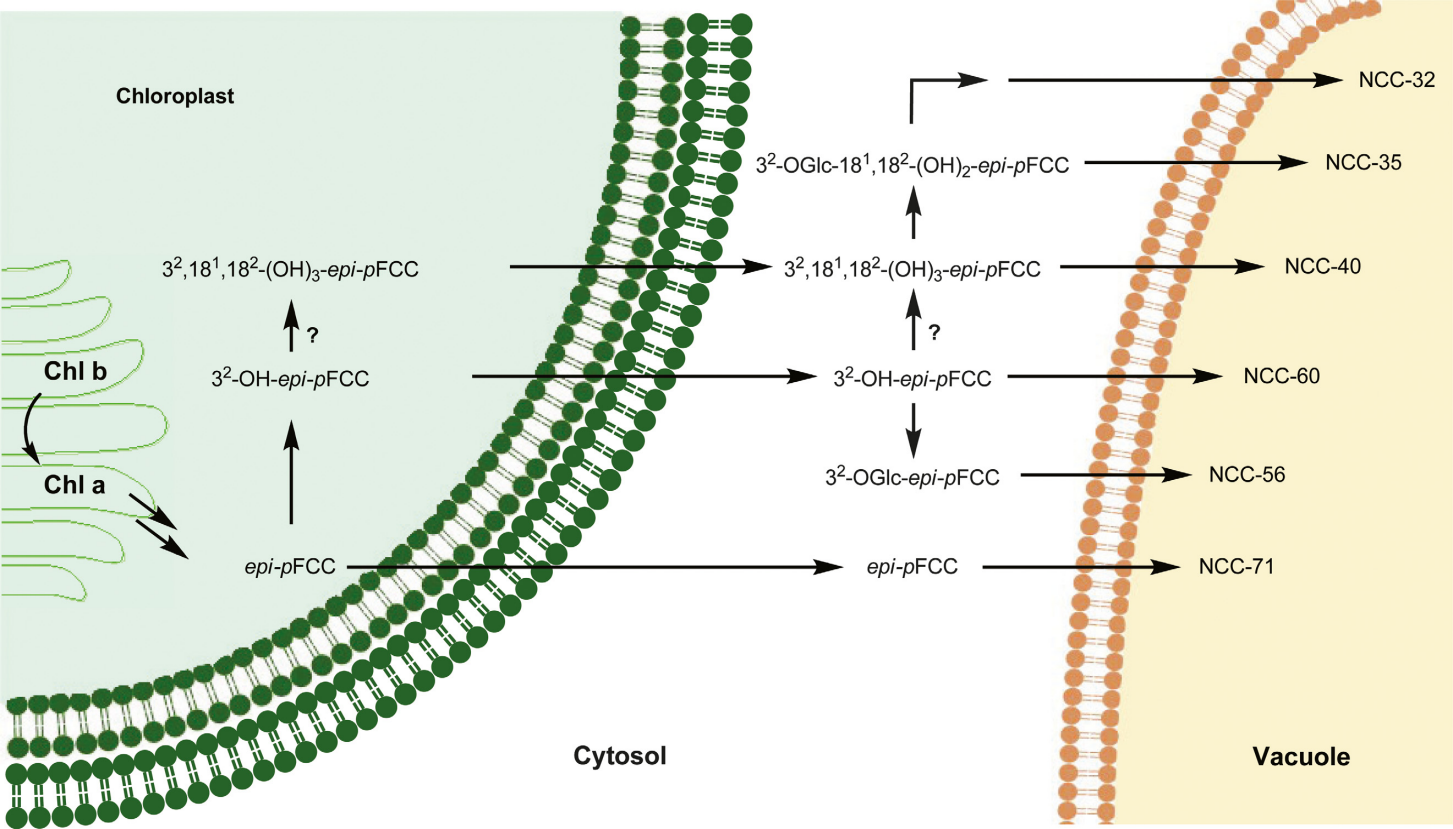
Hypothetical steps of chlorophyll (Chl) breakdown in senescent leaves of the plum tree (*Prunus domestica* ssp. *domestica*) in a topographical model, highlighting the major catabolic steps with abridged short names of (hypothetical) fluorescent chlorophyll catabolites (FCC) intermediates and of non‐fluorescent chlorophyll catabolites (NCCs), characterized in the present work.

Extracts of naturally senescent leaves of the plum tree (*Prunus domestica* ssp. *domestica*) were shown to contain a range of NCCs, two YCCs, and, in traces, a PiCC, all members of the ‘type I’ phyllobilin family. In spite of the absence of DCCs,^7^, ^8^ a remarkable structural diversity of Chl‐catabolites was, thus, indicated. The polar NCC *Pd*‐NCC‐32 (**1**) showed a previously unknown structure and is functionalized with two glycopyranose moieties on the ‘distant’ pyrrole rings *A* and *D*. The structure of *Pd*‐NCC‐32 (**1**) also provided the first (indirect) evidence for enzymatic glycosidation of an FCC at the 18^2^‐position (a primary alcohol function resulting from dihydroxylation of the corresponding vinyl group of the precursor FCC).^7^, ^8^ Five more NCCs were tentatively identified with known catabolites based on their matching UV/VIS‐ and mass spectroscopic features. Further identification by HPLC of *Pd*‐NCC‐60 (**5**) and *Pd*‐NCC‐71 (**6**) with corresponding *Cj*‐NCCs, indicated the plum NCCs to belong to the C(16)‐*epi* series, as well.^8^, ^9^, ^26^ Additional investigations will be required to secure the structures of several minor NCC‐ and of the YCC‐containing fractions. Based on the deduced structures of the plum NCCs, a tentative pathway of their formation in the senescent leaves of the plum tree could be derived (see *Fig.* 8).

While the first Chl‐catabolites in *Rosaceae* crops were found in leaves and fruits of apple and pear trees,^17^ which belong to the Pyreae tribus, here a stone fruit (that is part of the Amygdaleae tribus) was studied for the first time. The findings with senescent leaves of the plum tree are consistent with the related earlier studies with leaves of apple and pear trees.^17^ With members like apples, pears, peaches, strawberries, raspberries and many others, the *Rosaceae* family belongs to the six most economically important crop families worldwide.^43^ Thus, this study suggests the conserved PaO/phyllobilin pathway of Chl breakdown to NCCs to operate in senescent leaves of the Spiraeoideae subfamily of the *Rosaceae*.

## Experimental Section

### General

HPLC grade MeOH was purchased from *HiPerSolv Chromanorm* (Fontenay‐sous‐Bois, F), LC/MS gradient grade MeOH from or *VWR* (Milan, Italy), and AcONH_4_, puriss. p.a., from *Fluka* (Buchs, CH). KH_2_PO_4_, puriss. p.a., K_3_PO_4_ dibasic‐anh., puriss. p.a., and hexane were from *Sigma–Aldrich* (St. Louis, USA). Sand was from *J. T. Baker* (Avantor, PA, USA), *Sep‐Pak* ^®^ *C*_*18*_ cartridges (1 and 5 g) were from *Waters Associates*. pH Values were measured with a W*TW SenTix 21* electrode connected to a *WTW pH525* digital pH meter.

*HPLC. Dionex Summit* HPLC system with manual sampler, *P680* pump, online degasser and diode array detector, 1.35 ml or 200 μl injection loop. Data were collected and processed with Chromeleon V6.70.

*i*) *Anal. HPLC*. *Kinetex 00G‐4601‐E0‐5u‐C*_*18*_ *‐100A* 250 × 4.6 mm i.d. column at 20 °C protected with a *Phenomenex AJ0‐4287 C*_*18*_ 4 × 3.0 mm i.d. pre‐column was used with a flow rate of 0.5 ml min^−1^. Solvent *A*: 50mm aq. potassium phosphate buffer (pH 7.0), solvent *B*: MeOH, solvent *C*: H_2_O; solvent composition (*A*/*B*/*C*) as a function of time (0 – 90 min): 0 – 5, 80:20:0; 5 – 60, 80:20:0 to 40:60:0; 60 – 80, 40:60:0 to 0:100:0; 80 – 85, 0:100:0; 85 – 87, 0:100:0 to 0:20:80; 87 – 90, 0:20:80 to 80:20:0.

*ii*) *Semi‐prep. HPLC (90* *min run)*. *00G‐4252‐NO Luna 5u C*_*18*_ *(2) 100A* 250 × 10 mm i.d. column at 20 °C protected with a *Phenomenex AJ0‐7220* 250 × 10 mm i.d. pre‐column was used with a flow rate as a function of time: 0 – 5 min: 1 – 4 ml min^−1^; 5 – 90 min: 4 ml min^−1^. Solvent *A*: 4mm aq. AcONH_4_, solvent *B*: MeOH with AcONH_4_ (*c* = 4mm), solvent *C*: H_2_O; solvent composition (*A*/*B*/*C*) as a function of time (0 – 90 min): 0 – 5, 80:20:0; 5 – 60, 80:20:0 to 40:60:0; 60 – 80, 40:60:0 to 0:100:0; 80 – 85, 0:100:0; 85 – 87, 0:100:0 to 0:20:80; 87 – 90, 0:20:80 to 80:20:0.

*iii*) *Semi‐prep. HPLC (70* *min run)*. *00G‐4252‐NO Luna 5u C*_*18*_ *(2) 100A* 250 × 10 mm i.d. column at 20 °C protected with a *Phenomenex AJ0‐7220/1 C*_*18*_ 250 × 10 mm i.d. pre‐column was used with a flow rate as a function of time: 0 – 5 min: 1 – 4 ml min^−1^; 5 – 70 min: 4 ml min^−1^. Solvent *A*: 50mm aq. potassium phosphate buffer (pH 7.0), solvent *B*: MeOH, solvent *C*: H_2_O; solvent composition (*A*/*B*/*C*) as function of time (0 – 70 min): 0 – 5, 80:20:0; 5 – 50, 80:20:0 to 47.3:52.7:0; 50 – 55, 47.3:52.7:0 to 0:52.7:47.3; 55 – 60, 0:52.7:47.3 to 0:100:0; 60 – 65, 0:100:0; 65 – 67, 0:100:0 to 0:20:80; 67 – 70, 0:20:80 to 80:20:0.

*LC/MS. i*) *Pre‐Purifcation of Minor Fractions Pd*‐NCC‐54 and *Pd*‐NCC‐71 *on an anal*. *HPLC*. Minor catabolite fractions were first purified by HPLC (*Agilent 1260 Infinity*;* Agilent Technologies*, Santa Clara, California, USA) according to following procedure: 3 – 4 g of leaf material were ground in mortar and pestle under liquid N_2_ with addition of *ca*. 1 g of sand, a tip of a spatula of CaCO_3_ and 4 – 5 ml of MeOH. The mixture was centrifuged (6 min, 7200 *g*, 4 °C) and the supernatants were stored at −80 °C until use. An aliquot of the supernatant was centrifuged (1 min at 7200 *g*), diluted (1:1 *v*/*v*) with aq. potassium phosphate buffer (50mm, pH 7.0) and centrifuged again (1 min at 7200 *g*, 4 °C). In total 300 μl (3 × 100 μl) of the supernatant were purified on the anal. HPLC (pre‐column: *Phenomenex SecurityGuard Cartridge C*_*18*_, 4 × 3 mm; column: *Phenomenex HyperClone* 5 μm, *ODS C*_*18*_ *120A*; 250 × 4.6 mm; column temp., 20 °C) at a flow rate of 0.5 ml min^−1^ using 50mm aq. K_3_PO_4_ as solvent *A* and MeOH as solvent *B* (0 – 110 min: 0 – 5, 80:20; 5 – 80, 80:20 to 30:70; 80 – 85, 30:70 to 0:100; 85 – 95, 0:100; 95 – 100, 0:100 to 80:20; 100 – 110, 80:20), and desired fractions were collected and combined.

*ii*) LC/MS Analysis of minor fractions of the collected HPLC fractions were analyzed on an LC/MS system (*Thermo Fisher*,* Accela 1250* pump, *Accela PDA* detector, TSQ Quantum Access Max) using AcONH_4_ buffer (4mm, solvent *A*) and MeOH (LC/MS gradient grade, solvent *B*) as eluents (pre‐column: *Phenomenex Security Guard Cartridge C*_*18*_, 4 × 3 mm; Column: *Phenomenex HyperClone* column, 5 μm, *ODS C*_*18*_ *120A*; 250 × 4.6 mm; column temp., 25 °C). Twenty microliter of the collected catabolite fraction were injected and analyzed at a flow rate of 0.5 ml min^−1^ (0 – 57 min: 0 – 5, 80:20; 5 – 30, 80:20 to 30:70; 30 – 35, 30:70 to 0:100; 35 – 50, 0:100; 50 – 51, 0:100 to 80:20; 51 – 57, 80:20).

*Spectroscopy*. UV/VIS Spectra: *Agilent Technologies Cary 60* spectrophotometer; *λ*_max_ (nm) (rel. *ε*). CD Spectra: *Jasco J715*,* λ*_max_ and *λ*_min_ (nm), Δ*ε*. ^1^H‐ and ^13^C‐NMR: *Bruker 600 MHz Avance II+* (*δ*(C^1^HD_2_OD) 3.31 ppm, and *δ*(^13^CD_3_OD) 49.0 ppm, *δ* in ppm,^44^ *J* in Hz. Mass Spectrometry: *Finnigan LCQ Classic*, electrospray ionization (ESI) source, positive‐ion mode,^42^ 4.5 kV spray voltage (rel. abundance).

### Analysis of Chl‐Catabolites in Senescent Leaves by HPLC

Senescent plum tree leaves were harvested in November 2013 from a commercial orchard in Aldino (South Tyrol). They were immediately frozen in a freezer (−80 °C) and transported in a cold box (−20 °C) to Innsbruck, where they were stored cold (−80 °C).

A leaf segment (with the area of about 20 cm^2^) was frozen in liquid N_2_, grounded in a mortar and extracted with 1 ml of MeOH. The resulting suspension was centrifuged for 3 min at 13,000 *g*. Five hundred microliter of the MeOH supernatant were diluted with 2 ml of 50mm aq. potassium phosphate buffer (pH 7.0). After centrifugation for 3 min at 13,000 *g*, 200 μl of the extract was analyzed by HPLC (see *Fig.* 2).

*Isolation and Structure Elucidation of Pd*‐NCC‐32 (**1**). Yellow‐greenish senescent plum tree leaves (18.7 g) were frozen in liquid N_2_, pulverized to a fine powder and extracted with 60 ml of MeOH. The suspension was centrifuged for 5 min at 4000 *g*. Forty‐two milliliter of the supernatant were diluted with 168 ml of 50mm aq. potassium phosphate buffer (pH 7.0). After centrifugation for 5 min at 4000 *g*, the soln. was extracted two times with hexane. The MeOH extract was diluted with 300 ml of 50mm potassium phosphate buffer (pH 7.0) and applied to a pre‐conditioned 5 g *SepPak* cartridge. This was washed with 35 ml of H_2_O and the NCC‐containing fraction was eluted with 30 ml of MeOH. The solvents were removed by using a rotary evaporator. The residue was dissolved in 1 ml of MeOH and 4 ml of 50mm aq. potassium phosphate buffer (pH 7.0) using an ultrasonic bath. After centrifugation for 3 min at 13,000 *g*, the sample was divided in four aliquots and applied to semi‐prep. HPLC; injection volume, 1.25 ml; flow rate, 0 – 5 min: 1 – 4 ml min^−1^, 5 – 90 min: 4 ml min^−1^; solvent *A*: 4mm aq. AcONH_4_, solvent *B*: MeOH with AcONH_4_ (*c* = 4mm), solvent *C*: H_2_O; solvent composition (*A*/*B*/*C*) as a function of time (0 – 90 min): 0 – 5, 80:20:0; 5 – 60, 80:20:0 to 40:60:0; 60 – 80, 40:60:0 to 0:100:0; 80 – 85, 0:100:0; 85 – 87, 0:100:0 to 0:20:80; 87 – 90, 0:20:80 to 80:20:0. Fractions containing *Pd*‐NCC‐32 (**1**) of five consecutive semi‐prep. HPLC runs were collected and dried under reduced pressure. The residue was dissolved in 200 μl of MeOH and 800 μl of 50mm aq. potassium phosphate buffer (pH 7.0) and re‐purified by semi‐prep. HPLC; injection volume, 1.00 ml; flow rate, 0 – 5 min: 1 – 4 ml min^−1^, 5 – 70 min: 4 ml min^−1^; solvent *A*: 50mm aq. potassium phosphate buffer (pH 7.0), solvent *B*: MeOH, solvent *C*: H_2_O; solvent composition (*A*/*B*/*C*) as a function of time (0 – 70 min): 0 – 5, 80:20:0; 5 – 50, 80:20:0 to 47.3:52.7:0; 50 – 55, 47.3:52.7:0 to 0:52.7:47.3; 55 – 60, 0:52.7:47.3 to 0:100:0; 60 – 65, 0:100:0; 65 – 67, 0:100:0 to 0:20:80; 67 – 70, 0:20:80 to 80:20:0. The fraction containing *Pd*‐NCC‐32 (**1**) was collected between and diluted with 20 ml of 50mm aq. potassium phosphate buffer (pH 7.0). For de‐salting, the aq. soln. was applied to a pre‐conditioned 5 g *SepPak* cartridge, washed with 15 ml of H_2_O and eluted with 5 ml of MeOH. After removal of the solvents using a rotary evaporator, the sample was dried under high vacuum and a uniform sample of 0.29 mg of *Pd*‐NCC‐32 (**1**) was obtained.

*Isolation of Raw Pd*‐NCCs *for Structural Analysis*. 12 anal. extracts were prepared, combined and diluted with 95 ml of 50mm aq. potassium phosphate buffer (pH 7.0). This was applied to a pre‐conditioned 5 g *SepPak* cartridge, washed with 30 ml of H_2_O and the NCC‐containing fraction was eluted with 30 ml of MeOH. The fraction was dried under reduced pressure and the precipitate was dissolved in 400 μl of MeOH and 1.6 ml of 4mm aq. AcONH_4_. After centrifugation for 3 min at 13,000 *g*, the sample was divided in two aliquots and applied to semi‐prep. HPLC; injection volume, 1.00 ml; flow rate, 0 – 5 min: 1 – 4 ml min^−1^, 5 – 90 min: 4 ml min^−1^; solvent *A*: 4mm aq. AcONH_4_, solvent *B*: MeOH with AcONH_4_ (*c* = 4mm), solvent *C*: H_2_O; solvent composition (*A*/*B*/*C*) as a function of time (0 – 90 min): 0 – 5, 80:20:0; 5 – 60, 80:20:0 to 40:60:0; 60 – 80, 40:60:0 to 0:100:0; 80 – 85, 0:100:0; 85 – 87, 0:100:0 to 0:20:80; 87 – 90, 0:20:80 to 80:20:0. The fractions containing *Pd*‐NCC‐32 (**1**), *Pd*‐NCC‐35 (**2**), *Pd*‐NCC‐40 (**3**), *Pd*‐NCC‐56 (**4**) and *Pd*‐NCC‐60 (**5**) were collected and to obtain pure samples from all fractions an anal. HPLC run with AcONH_4_ had to be performed; injection volume, 200 μl; flow rate, 0.5 ml min^−1^; solvent *A*: 4mm aq. AcONH_4_, solvent *B*: MeOH with AcONH_4_ (*c* = 4mm), solvent *C*: H_2_O; solvent composition (*A*/*B*/*C*) as a function of time (0 – 90 min): 0 – 5, 80:20:0; 5 – 60, 80:20:0 to 40:60:0; 60 – 80, 40:60:0 to 0:100:0; 80 – 85, 0:100:0; 85 – 87, 0:100:0 to 0:20:80; 87 – 90, 0:20:80 to 80:20:0. In each anal. HPLC run, the desired catabolite was collected.

### Spectroscopic data (for atom numbering)

**Figure 9:**
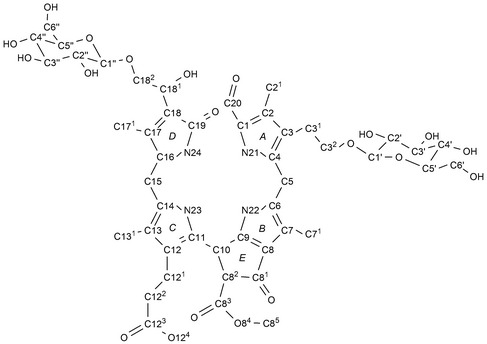
Atom numbering and labels of rings, used for *Pd*‐NCC‐32 (**1**), representative of numbering used for other non‐fluorescent chlorophyll catabolites (NCCs).

***Pd*‐NCC‐32** (**1**). *t*_R_ = 32.6 min. UV/VIS (MeOH, *c* = 4.3 × 10^−5^ m): 244sh (0.83), 314 (1.00) (see *Fig*. 9). CD (MeOH, *c* = 4.3 × 10^−5^ m): 226 (8), 249 (−3), 263 (−3), 283 (−8), 319 (1). ^1^H‐NMR (600 MHz, CD_3_OD, 10 °C): 1.92 (*s*, Me(13^1^)); 2.08 (*s*, Me(17^1^)); 2.12 (*s*, Me(7^1^)); 2.23 (*s*, Me(2^1^)); 2.26 – 2.30 (*m*, H_a_–C(12^2^)); 2.31 – 2.35 (*m*, H_b_–C(12^2^)); 2.37 – 2.41 (*m*, H_a_–C(15)); 2.60 – 2.65 (*m*, CH_2_(3^1^), H_a_–C(12^1^)); 2.71 – 2.77 (*m*, H_b_–C(12^1^)); 2.91 (*dd*,* J* = 4.0, 14.6, H_b_–C(15)); 3.17 (*dd*,* J* = 7.8, 9.2, H–C(2′)); 3.21 (*dd*,* J* = 7.8, 9.2, H–C(2″)); 3.24 – 3.28 (*m*, H–C(4′), H–C(4″), H–C(5′), H–C(5″)); 3.33 – 3.39 (*m*, H_a_–C(3^2^), H–C(3′), H–C(3″)); 3.62 – 3.69 (*m*, H_a_–C(6′), H_a_–C(6″), H_b_–C(3^2^)); 3.71 (*dd*,* J* = 5.8, 12.0, H_a_–C(18^2^)); 3.75 (*s*, Me(8^5^)); 3.83 – 3.87 (*m*, H_b_–C(6′), H_b_–C(6″)); 3.96 – 4.01 (*m*, H_a_–C(5), H_b_–C(18^2^)); 4.02 – 4.09 (*m*, H–C(16), H_b_–C(5)); 4.17 (*d*,* J* = 7.8, H–C(1′)); 4.33 (*d*,* J* = 7.8, H–C(1″)); 4.77 (*dd*,* J* = 3.5, 7.6, H–C(18^1^)); 4.87 (*s*, H–C(10)); 9.32 (*s*, H–C(20)). ^13^C‐NMR (^13^C‐signal assignment from ^1^H,^13^C‐HSQC and ^1^H,^13^C‐HMBC experiments in CD_3_OD, 10 °C): 8.4 (C(2^1^)); 9.1 (C(7^1^)); 9.1 (C(13^1^)); 12.5 (C(17^1^)); 22.0 (C(12^1^)); 23.6 (C(5)); 24.8 (C(3^1^)); 29.7 (C(15)); 36.9 (C(10)); 39.6 (C(12^2^)); 52.7 (C(8^5^)); 62.1 (C(6′)); 62.1 (C(6″)); 62.5 (C(16)); 66.6 (C(18^1^)); 67.7 (C(8^2^)); 70.3 (C(3^2^)); 71.1 (C(4′)); 71.1 (C(4″)); 73.2 (C(18^2^)); 74.8 (C(2′)); 74.8 (C(2″)); 77.7 (C(3′)); 77.7 (C(3″)); 77.7 (C(5′)); 77.7 (C(5″)); 103.9 (C(1′)); 103.9 (C(1″)); 112.2 (C(7)); 114.8 (C(13)); 120.0 (C(3)); 120.0 (C(12)); 124.2 (C(11)); 124.2 (C(14)); 125.6 (C(8)); 128.5 (C(1)); 133.9 (C(6)); 134.0 (C(2)); 139.6 (C(4)); 159.3 (C(17)); 130.2 (C(18)); 161.5 (C(9)); 171.4 (C(8^3^)); 174.8 (C(19)); 177.2 (C(20)). ESI‐MS: 1079.2 (6, [*M* − H + 2K]^+^); 1063.3 (12, [*M* − H + K + Na]^+^); 1047.3 (6, [*M* − H + 2Na]^+^); 1041.3 (57, [*M* + K]^+^); 1025.3 (73, [*M* + Na]^+^); 1005.1 (20), 1004.1 (53), 1003.1 (100, $C_{47}H_{63}N_{4}O_{20}^{+}$, [*M* + H]^+^); 987.3 (7); 879.3 (7, [*M* − C_6_H_10_O_5_ + K]^+^); 863.4 (8, [*M* − C_6_H_10_O_5_ + Na]^+^); 841.2 (36, [*M* − C_6_H_10_O_5_ + H]^+^); 827.3 (4); 684.1 (3, [*M* − C_7_H_11_NO_3_ − C_6_H_10_O_5_ + H]^+^); 679.2 (4, [*M* − 2 C_6_H_10_O_5_ + H]^+^).

***Pd*‐NCC‐35** (**2**). *t*_R_ = 35.5 min. UV/VIS (4mm aq. AcONH_4_/MeOH 63:37): 284 (0.75), 316 (1.00). ESI‐MS: 879.3 (15, [*M* + K]^+^); 863.3 (37, [*M* + Na]^+^); 843.2 (15), 842.2 (47), 841.2 (100, $C_{41}H_{53}N_{4}O_{15}^{+}$, [*M* + H]^+^); 825.3 (6); 809.3 (4, [*M* − CH_4_O + H]^+^); 684.2 (8, [*M* − C_7_H_11_NO_3_ + H]^+^); 679.2 (6, [*M* − C_6_H_10_O_5_ + H]^+^); 522.1 (1, [*M* − C_7_H_11_NO_3_ − C_6_H_10_O_5_ + H]^+^).

***Pd‐*NCC‐40** (**3**). *t*_R_ = 40.6 min. UV/VIS (4mm aq. AcONH_4_/MeOH 59:41): 278 (0.78), 316 (1.00). ESI‐MS: 755.1 (8, [*M* − H + 2K]^+^); 739.2 (7, [*M* − H + K + Na]^+^); 717.3 (71, [*M* + K]^+^); 701.3 (46, [*M* + Na]^+^); 681.2 (10), 680.1 (40), 679.2 (100, $C_{35}H_{43}N_{4}O_{10}^{+}$, [*M* + H]^+^); 647.2 (12, [*M* − CH_4_O + H]^+^); 522.1 (5, [*M* − C_7_H_11_NO_3_ + H]^+^).

***Pd*‐NCC‐54**. UV/VIS (4mm aq. AcONH_4_/MeOH 40:60): 316 nm. ESI‐MS: 699.2 (14, [*M* + K]^+^); 678.2 (16, [*M* + NH_4_]^+^); 663 (4), 662 (32), 661.2 (100, $C_{35}H_{41}N_{4}O_{9}^{+}$, [*M* + H]^+^); 629.3 (3, [*M* − CH_4_O + H]^+^).

***Pd‐*NCC‐56** (**4**). *t*_R_ = 56.4 min. UV/VIS (4mm aq. AcONH_4_/MeOH 47:53): 316 nm. ESI‐MS: 845.3 (23, [*M* + K]^+^); 829.3 (26, [*M* + Na]^+^); 809.2 (15), 808.2 (46), 807.2 (100, $C_{41}H_{51}N_{4}O_{13}^{+}$, [*M* + H]^+^); 775.3 (10, [*M* − CH_4_O + H]^+^); 684.2 (5, [*M* − C_7_H_9_NO + H]^+^); 645.2 (13, [*M* − C_6_H_10_O_5_ + H]^+^).

***Pd‐*NCC‐60** (**5**). *t*_R_ = 60.0 min. UV/VIS (4mm aq. AcONH_4_/MeOH 44:56): 315 (1.00). ESI‐MS: 683.2 (15, [*M* + K]^+^); 667.3 (22, [*M* + Na]^+^); 647.2 (11), 646.2 (39), 645.2 (100, $C_{35}H_{41}N_{4}O_{8}^{+}$, [*M* + H]^+^); 613.2 (19, [*M* − CH_4_O + H]^+^); 522.1 (8, [*M* − C_7_H_9_NO + H]^+^).

**Figure 10:**
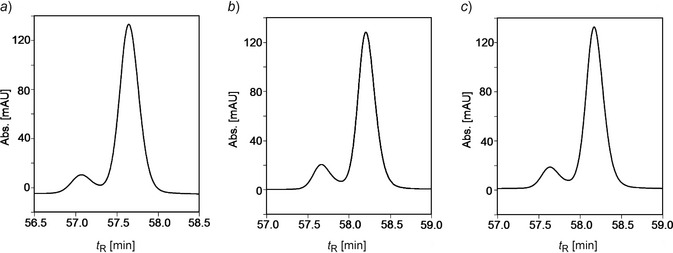
Identification of *Pd*‐NCC‐60 (**5**) with *Cj*‐NCC‐1 by HPLC. Samples *a*): of *Cj*‐NCC‐1; *b*): of *Pd*‐NCC‐60 (**5**); *c*): 1:1 mixture of *Pd*‐NCC‐60 (**5**) and *Cj*‐NCC‐1.

Identification of *Pd*‐NCC‐60 (**5**) and *Cj*‐NCC‐1^33^ by HPLC co‐injection experiment; separate samples of purified *Pd*‐NCC‐60 (**5**), of *Cj*‐NCC‐1, as well as a 1:1 mixture of both were analyzed by anal. HPLC (see *Fig.* 10).

***Pd‐*NCC‐71** (**6**). *t*_R_ = 70.7 min. UV/VIS (50mm aq. potassium phosphate buffer (pH 7.0)/MeOH 20:80): 239sh (1.00), 316 (0.87). ESI‐MS: 667.1 (4, [*M* + K]^+^); 631 (2), 630 (35), 629.2 (100, $C_{35}H_{41}N_{4}O_{7}^{+}$, [*M* + H]^+^); 597.2 (23, [*M* − CH_4_O + H]^+^); 506 (5, [*M* − C_7_H_9_NO + H]^+^). Provisional identification of *Pd*‐NCC‐71 (**6**) with *Cj*‐NCC‐2^13^ by HPLC co‐injection experiment; an extract of a plum tree leaf containing *Pd*‐NCC‐71 (**6**), a separate sample of purified *Cj*‐NCC‐2, as well as a mixture of both were analyzed by anal. HPLC.

***Pd*‐YCC‐61**. *t*_R_ = 61.3 min. UV/VIS (50mm aq. potassium phosphate buffer (pH 7.0)/MeOH 40:60): 246 (0.73), 313 (1.00), 429 (1.77). ESI‐MS: 881.1 (18, [*M* − H + 2K]^+^); 865.3 (14, [*M* − H + K + Na]^+^); 843.1 (52, [*M* + K]^+^); 827.3 (37, [*M* + Na]^+^); 807.2 (17), 806.1 (48), 805.1 (100, $C_{41}H_{49}N_{4}O_{13}^{+}$, [*M* + H]^+^); 796.6 (16); 774.4 (26); 756.3 (17); 700.3 (15); 643.1 (17, [*M* − C_6_H_10_O_5_ + H]^+^); 611.3 (5, [*M* − C_6_H_10_O_5_ − CH_4_O + H]^+^).

***Pd*‐YCC‐67**. *t*_R_ = 67.1 min. UV/VIS (50mm aq. potassium phosphate buffer (pH 7.0)/MeOH) 25:75): 247 (0.74), 316 (1.00), 428 (1.29). ESI‐MS: 719.1 (2, [*M* − H + 2K]^+^); 703.2 (8, [*M* − H + K + Na]^+^); 681.1 (12, [*M* + K]^+^); 665.3 (26, [*M* + Na]^+^); 645.2 (11), 644.2 (40), 643.2 (100, C_35_H_39_N_4_O_8_, [*M* + H]^+^); 611.2 (19, [*M* − CH_4_O + H]^+^).

***Pd*‐PiCC‐75**. *t*_R_ = 75.0 min. UV/VIS (50mm aq. potassium phosphate buffer (pH 7.0)/MeOH 1:9): 314 (0.75), 525 (1.00).

Provisional identification of *Pd*‐PiCC‐75 (in an extract of a plum tree leaf) and of a purified sample of *Cj*‐PiCC^29^ by HPLC (separate runs and co‐injection experiment).
